# Development of tannic acid-enriched materials modified by poly(ethylene glycol) for potential applications as wound dressing

**DOI:** 10.1007/s40204-020-00136-1

**Published:** 2020-09-20

**Authors:** Beata Kaczmarek, Olha Mazur, Oliwia Miłek, Marta Michalska-Sionkowska, Anna M. Osyczka, Konrad Kleszczyński

**Affiliations:** 1 Department of Biomaterials and Cosmetics Chemistry, Faculty of Chemistry, Nicolaus Copernicus University, Gagarina 7, 87-100 Toruń, Poland; 2 Department of Biology and Cell Imaging, Faculty of Biology, Institute of Zoology and Biomedical Research, Jagiellonian University, Gronostajowa 7, 30-387 Kraków, Poland; 3 Department of Environmental Microbiology and Biotechnology, Faculty of Biology and Environmental Protection, Nicolaus Copernicus University, Lwowska 1, 87-100 Toruń, Poland; 4 Department of Dermatology, University of Münster, Von-Esmarch-Str. 58, 48149 Münster, Germany

**Keywords:** Tannic acid, Poly(ethylene glycol), Regeneration, Wound dressing, Proliferation

## Abstract

The interests in the biomedical impact of tannic acid (TA) targeting production of various types of biomaterials, such as digital microfluids, chemical sensors, wound dressings, or bioimplants constantly increase. Despite the significant disadvantage of materials obtained from natural-based compounds and their low stability and fragility, therefore, there is an imperative need to improve materials properties by addition of stabilizing formulas. In this study, we performed assessments of thin films over TA proposed as a cross-linker to be used in combination with polymeric matrix based on chitosan (CTS), i.e. CTS/TA at 80:20 or CTS/TA at 50:50 and poly(ethylene glycol) (PEG) at the concentration of 10% or 20%. We evaluated their mechanical parameters as well as the cytotoxicity assay for human bone marrow mesenchymal stem cells, human melanotic melanoma (MNT-1), and human osteosarcoma (Saos-2). The results revealed significant differences in dose-dependent of PEG regarding the maximum tensile strength (*σ*_max_) or impact on the metabolic activity of tissue culture plastic. We observed that PEG improved mechanical parameters prominently, decreased the hemolysis rate, and did not affect cell viability negatively. Enclosed data, confirmed also by our previous reports, will undoubtedly pave the path for the future application of tannic acid-based biomaterials to treat wound healing.

## Introduction

Polymers have multiple capabilities to be used in targeting production of various types of biomaterials, for instance, digital microfluids, chemical sensors, wound dressings, or bioimplants (Jayaprakash et al. 2015; Kang et al. 2011; Sarvothaman et al. 2015). They are known to possess excellent film-forming abilities and are miscible with different types of synthetic and natural compounds (Lewandowska et al. 2016). Biopolymers exert high biocompatibility and show adverse effects, thereby they are commonly applied to obtain wide types of materials for their biomedical purposes. Nevertheless, the significant disadvantage of materials obtained from natural-based compounds and their low stability and fragility (Bassyouni et al. 2017; Kamoun et al. 2017; Pandit et al. 2018); therefore, there is an imperative to introduce to improve materials’ properties by addition of stabilizing compounds (Kaczmarek et al. 2017; Lee et al. 2018; Lima et al. 2018a, b).

Polyphenols (as tannic acid) are natural compounds recently considered for the wide medical applications. They may act as polysaccharides cross-linkers that consist of many hydroxyl (–OH) groups in the structure, thereby they are able to interact with hydrophilic functional groups from polymeric chain and form strong hydrogen interactions. For instance, tannic acid has been studied concomitantly with chitosan (Kaczmarek et al. 2019a). To date, mainly phenolic acids are used as additives to polymeric materials, such as ferulic acid (Cirillo et al. 2018), ellagic acid (Karaseva et al. 2019), gallic acid (Xie et al. 2018) as well as tannic acid (Buono et al. 2018). They also provide new active biological properties as antibacterial or antioxidant (de Dicastillo et al. 2016; Kadzinska et al. 2019). Moreover, scaffolds containing tannic acid were implanted into the rabbits’ bone where the healing processes were significantly elevated (Gentile et al. 2017). The presence of tannic acid also has improved the blood vessels formation what is essential for tissue regeneration. The blood compatibility of polymeric material is the key feature that determines its application in contact with blood. It should be tested whether the presence of material causes the hemolysis. If the hemolysis exceeds 5%, material cannot be applied as wound dressing. It should be emphasized that water vapor permeability is an important factor in materials’ research. It allows maintaining moisturized environment around the healing wound. Thus, dry conditions may delay the healing processes and are unwanted while wound dressing materials are considered. The results presented recently by Kaczmarek et al. (2018) prompted us to elucidate the potential role of tannic acid in biomedicine and regenerative medicine. Herein, we additionally propose poly(ethylene glycol) (PEG) as a modifier that improves its properties. Thus, it was earlier presented that additives, such as cellulose (Faradilla et al. 2019) collagen/chitosan (Kozłowska et al. 2018), or sodium alginate (Sun et al. 2019), exert developing capacities of designed biomaterials. Herein, our results confirm the enhancement of materials’ physicochemical properties after the addition of PEGs emphasizing that its supplement as a stabilizer component for chitosan/tannic acid films still remain as not entirely described issue. The aim of the study is the novel approach regarding preparation and characterization of materials based on chitosan with tannic acid and poly(ethylene glycol) bringing the new insights into the future treatment of wound healing. On one side, we present the results of maximum tensile strength, water vapor permeation rate assessment and we elucidate the material properties in contact with blood determined as hemolysis, and platelet adhesion studies on the other side. We tested three human cell lines including bone marrow mesenchymal stem cells (BMSC), melanotic melanoma (MNT-1), and osteosarcoma (Saos-2). Altogether with previous reports listed above, enclosed results can be principal studies for further investigations of chitosan/tannic acid-based materials modified by PEG addition for wound dressings.

## Materials and methods

### Reagents

Dulbecco’s Modified Eagle’s Medium (DMEM) with high glucose (4500 mg/L), Minimal Essential Medium Eagle (MEM), 1% penicillin–streptomycin solution (10,000 units of penicillin and 10 mg of streptomycin in 1 mL 0.9% NaCl), acetic acid, calcium chloride, chitosan (CTS) (DD 77%), ethanol (EtOH), glutaraldehyde, HEPES (1 M), non-essential amino acids (NEAA) (100 ×), poly(ethylene glycol) (PEG); PEG1: high molecular weight (Mv 8000 g/mol), PEG2: low molecular weight (Mv 200 g/mol), sodium pyruvate (1 mM), and tannic acid (TA) were purchased from Sigma (St. Louis, MO, USA). Fetal bovine serum, 0.05% trypsin/0.53 mM EDTA solution, 1 × PBS (pH 7.4), l-glutamine (200 mM) were supplied by Thermo Fisher Scientific (Waltham, MA, USA).

### Samples preparation

CTS and TA were dissolved separately to the final concentration of 2% (w/v) in 0.1 M acetic acid while PEG was prepared in dd H_2_O (final concentration: 1% by w/v). Subsequently, CTS and TA were mixed in different proportions, i.e. 80:20 or 50:50, along the previous studies (Kaczmarek et al. 2019b), and then 10% and 20% (w/w) of PEG solutions were added based on CTS content as film modifier. Afterwards, the prepared mixture was poured on the plastic holder (10 cm × 10 cm) to allow evaporating the solvent. Tested materials were sterilized prior to cell biological characterization in the form of thin films at the bottom of tissue culture plastic (TCP) in presence of 75% EtOH for 10 min followed by rinsing twice using sterile 1 × PBS (pH 7.4) to remove remnants of alcohol.

### Cell culture

Human melanoma MNT-1 cell line was cultured in DMEM medium supplemented with 10% (v/v) heat-inactivated fetal bovine serum, 1% (v/v) l-glutamine, 1% (v/v) HEPES, 1% (v/v) NEAA, 1% (v/v) sodium pyruvate, 1% (v/v) streptomycin–penicillin solution. BMSC and Saos-2 cells were maintained in MEM medium supplemented with 10% (v/v) heat-inactivated fetal bovine serum and 1% (v/v) streptomycin–penicillin solution. All cell lines in the logarithmic growth phase were used prior to the assessments.

### Maximum tensile strength

Mechanical properties are very important for the materials as thin films. Thus, the maximum tensile strength (*σ*_max_) was determined for each type of film by the Z.05 testing machine (Zwick/Roell, Germany) with the initial force 0.1 MPa and the velocity of 5 mm/min. To perform mechanical testing, samples were immersed for 2 h in 1 × PBS (pH 7.4) prior to the measurement.

### Water vapor permeation rate (WVPR)

The WVPR of films was investigated using the method described earlier with slight modifications (Phaechamud et al. 2016). Briefly, calcium chloride as a desiccant was placed into the plastic container (40 mm diameter) and dried out for 24 h at 105 °C prior to further use (*m*_0_). Films were placed on top of the containers, sealed tightly, and compared afterwards with the containers without covers considered as control samples. After 24 h at 37 °C (*m*_t_), the samples were removed and the weight gain was determined as described before (Michalska-Sionkowska et al. 2018) along the enclosed formula:

1 $${\text{the}}\;{\text{percentage}}\;{\text{weight}}\;{\text{gain}}\;{\text{of}}\;{\text{CaCl}}_{2} = \frac{{m_{{\text{t}}} - m_{0} }}{{m_{0} }} \times 100{\text{\% }}.$$

### Blood compatibility

Blood compatibility assignment was prepared using contact methods as described earlier by Zhou et al. (2011). Anti-coagulated sheep blood (0.2 mL) was added to 10 mL of physiological saline solution containing different specimens (1 cm^2^). Positive ([OD]_positive_) and negative ([OD]_negative_) samples were prepared by adding 0.2 mL of fresh blood to water and physiological saline, respectively, and tubes were incubated at 37 °C for 1 h. Next, the suspension was centrifuged at 1000 rpm for 10 min, and the absorbance of the supernatant was measured by the microplate reader Multiscan FC (Thermo Fisher Scientific, Waltham, USA) at *λ* = 540 nm. Hemolysis rate was calculated using the equation:

2 $${\text{rate}}\;{\text{of}}\;{\text{hemolysis}}\;{\text{(\% )}} = \frac{{\left[ {{\text{OD}}} \right]{\text{specimen}} - \left[ {{\text{OD}}} \right]{\text{negative}}}}{{\left[ {{\text{OD}}} \right]{\text{positive}} - \left[ {{\text{OD}}} \right]{\text{negative}}}} \times 100{\text{\% }}.$$

### Tannic acid release

Materials were immersed in simulated body fluid (SBF; pH 7.4). Tannic acid concentration was determined by the Folin–Ciocalteu method with the use of standard curve. Briefly, the Folin–Ciocalteu (0.5 mL) reagent was mixed with Na_2_CO_3_ (1 mL), sample (1 mL), and distilled water to the final volume of 10 mL. The solution was then stored in 40 °C for 30 min and assessed by the UV–Vis spectrophotometer (UV-1800, Shimadzu, Switzerland) at *λ* = 725 nm to determine the concentration of released tannic acid.

### Platelet adhesion studies

For preparing platelet-rich plasma (PRP), the centrifugation of blood was conducted at 1000 rpm for 10 min. The samples were sterilized and equilibrated in 1 × PBS for 6 h at room temperature (RT). Subsequently, PBS was replaced with PRP and samples were incubated further at 37 °C for 90 min. Afterwards, the samples were washed three times with 3 mL 1 × PBS to remove the non-adherent platelets, and the adhered platelets were fixed in 2.5% glutaraldehyde for 60 min. Then, samples were washed again with 1 × PBS followed by dehydration in gradient ethanol–water solutions (15 min each), and imaged using the scanning electron microscope (LEO Electron Microscopy Ltd, England).

### Establishing cell cultures on the experimental films

BMSC were obtained from a 56-year-old male patient along the protocol (Institutional Review Board; No. 1072.6120.254.2017). BMSC, MNT-1 and Saos-2 cells were seeded directly onto material films or tissue culture plastic at a density of 1 × 10^4^/cm^2^ in 1 mL of culture medium exchanged every 48 h. The cell proliferation MTS assay was carried out for 6 days. Briefly, cells were rinsed once with 1 × PBS and further maintained in respective culture medium mixed with MTS reagent (Cell Titer 96^®^ AQ_ueous_ One Solution Cell Proliferation Assay, Promega, Madison, WI, USA) (1:10) in the final volume of 200 μL/well, and cultured in a humidified atmosphere of 5% CO_2_ at 37 °C. The reactions were developed until color change of the MTS reagent in culture wells versus control sample (cell-free well). Next, the MTS solutions were transferred to individual wells into 96-well plates and absorbance was measured at *λ* = 492 nm using a microplate reader (SpectraMax iD3 Molecular Devices, San Jose, CA, USA).

### Statistical analysis

Experiments were performed at least three times, with results expressed in each case as the mean + standard deviation (SD). Significant differences between results were determined by the univariate analysis of variance (ANOVA) or the Student’s *t* test and appropriate post hoc analysis using GraphPad Prism 7.05 software (La Jolla, CA, USA). Obtained data were normalized and are presented as percentage of the control sample. A *P* value of less than 0.05 was considered statistically significant.

## Results

### Mechanical properties

**Fig. 1:**
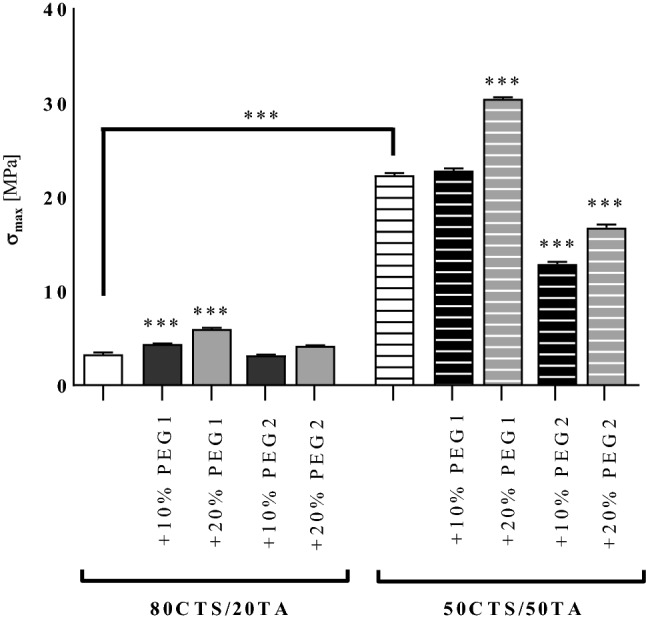
The maximum tensile strength (*σ*_max_) was evaluated in the films containing chitosan (CTS) or tannic acid (TA) in the weight ratio 80/20 and 50/50 with addition of PEG1 or PEG2 (w/w), and investigated as described in “Materials and methods”. Data were presented as the mean (*n* = 5) + SD. Statistically significant differences were indicated as *P < 0.05, **P < 0.01, ***P < 0.001

The maximum tensile strength values (*σ*_max_) for chitosan/tannic acid films without PEGs equaled to 3.27 MPa and 22.31 MPa (*P* < 0.001) for CTS/TA at 80:20 and CTS/TA at 50:50, respectively Fig. 1. Furthermore, two PEG concentrations were tested where *σ*_max_ was significantly elevated (*P* < 0.001) in case of CTS/TA at 80:20 + 10% PEG1 and 20% PEG1 compared to CTS/TA at 80:20 alone, reaching the value of 4.38 and 5.98, respectively. Similar pattern of regulation was observed in case of CTS/TA at 50:50. The presence of 20% PEG1 enhanced prominently (*P* < 0.001) *σ*_max_ compared to CTS/TA at 50:50 alone reaching the value of 30.46 for CTS/TA at 50:50 + 20% PEG1. On the other hand, the addition of PEG2 did not enhance the mechanical properties of films neither in case of CTS/TA at 80:20 nor CTS/TA at 50:50.

### Water vapor permeation rate (WVPR) and blood compatibility

Enclosed WVPR parameters for all assessed combinations of CTS/TA at 80:20 or CTS/TA at 50:50 were listed in Table 1. In our case, the WVPR of proposed films based on chitosan or tannic acid modified by PEGs ranged as follows: 4143 (CTS/TA at 80:20 + 20% PEG2) and 4498 (CTS/TA at 80:20 alone) or 3499 (CTS/TA at 50:50 + 20% PEG2) and 5697 g/m^2^/h (CTS/TA at 50:50 alone). It should be added that the control sample (container without film) reached the WVPR of 8390 g/m^2^/h. On the other hand, the addition of PEGs, either PEG1 or PEG2, triggers decrease of WVPR as seen in Table 1; however, the decrease is lower in case of PEG1 than PEG2.

**Table 1:** The water vapor permeation rate (WVPR) of films based on chitosan (CTS) or tannic acid (TA) in ratio 80/20 and 50/50 with addition of PEG1 or PEG2 (w/w) was assessed as described in “Materials and methods”

Specimen	WVPR (g/m^2^/h)
Container without film (control)	8390
80 CTS/20 TA	4498
+ 10% PEG1	4317
+ 20% PEG1	4209
+ 10% PEG2	4278
+ 20% PEG2	4143
50 CTS/50 TA	5697
+ 10% PEG1	4587
+ 20% PEG1	3551
+ 10% PEG2	4537
+ 20% PEG2	3499

Second, the PEG addition increased hemocompatibility of our materials compared to CTS/TA at 80:20 or CTS/TA at 50:50 alone (Table 2). Our experiment showed films with 10% PEG1 of their rate of hemolysis at 0.20% for CTS/TA at 80:20 mixtures or 0.09% for CTS/TA at 50:50. Similar trend was observed from PEG2 where 10% addition showed the rate of hemolysis 0.71% for CTS/TA at 80:20 specimen and 0.32% for CTS/TA at 50:50. Also, hemolysis rate slightly decreased with increasing PEG2 content. Thus, all the tested films with PEG addition showed the hemolysis rate below 1% indicating that materials are non-hemolytic.

**Table 2:** The rate of hemolysis, referred to blood compatibility, for the samples containing chitosan (CTS) or tannic acid (TA) in ratio 80/20 and 50/50 with addition of PEG1 or PEG2 (w/w) was evaluated as described in “Materials and methods”

Specimen	Rate of hemolysis (%)
80 CTS/20 TA	1.86 ± 0.57
+ 10% PEG1	0.20 ± 0.06
+ 20% PEG1	0.18 ± 0.03
+ 10% PEG2	0.71 ± 0.11
+ 20% PEG2	0.62 ± 0.09
50 CTS/50 TA	3.78 ± 0.08
+ 10% PEG1	0.09 ± 0.03
+ 20% PEG1	0.08 ± 0.05
+ 10% PEG2	0.32 ± 0.07
+ 20% PEG2	0.30 ± 0.02

### Tannic acid release and platelet adhesion studies

**Fig. 2:**
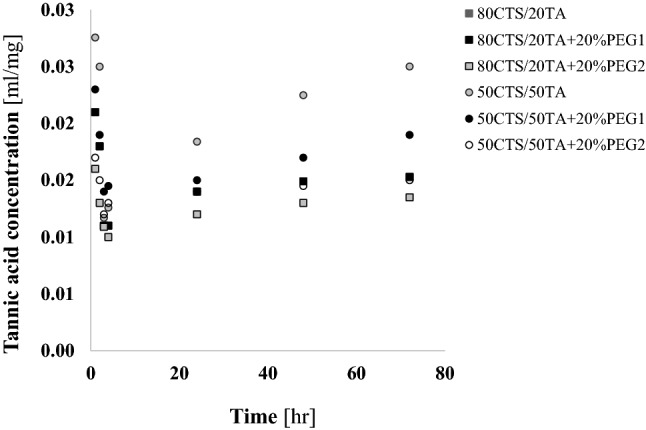
The tannic acid released concentration from CTS/TA at 80:20 or CTS/TA at 50:50 with and without 20% PEG1 or 20% PEG2 addition (w/w) was investigated as described in “Materials and methods”. Data were presented as the mean (*n* = 3)

The concentration of tannic acid released was detected after films’ immersion in three simulated body fluid (SBF; pH 7.4) and depicted in Fig. 2. The burst effect was noticed in the first period of time. Then, concentration of released tannic acid decreases for each type of film, and after 4 h of immersion the increase of tannic acid concentration was noticed resulting of films welling. As a consequence, regular increase of phenolic acid concentration was observed indicating that presence of PEG massively decreases tannic acid release what is seen particularly after extended incubation time (48 or 72 h).

**Fig. 3:**
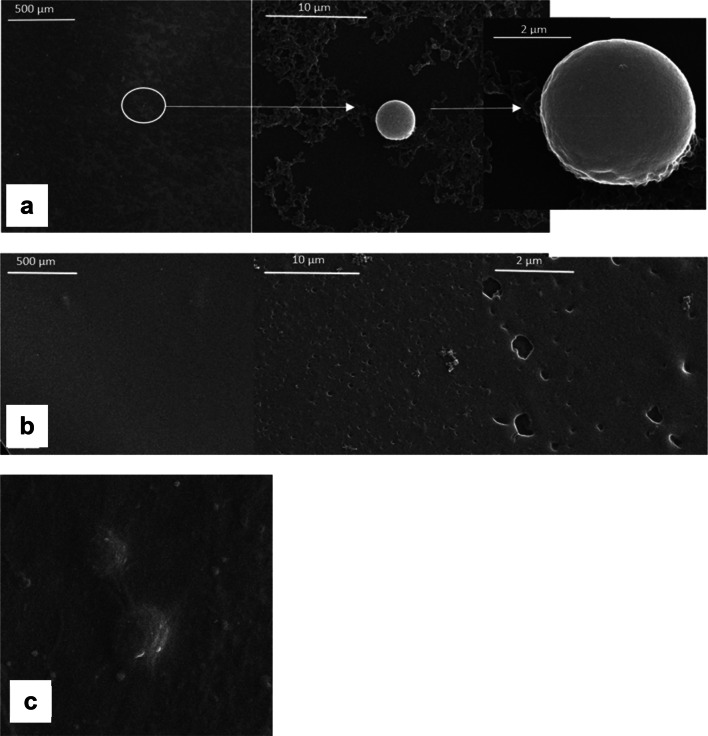
Platelet adhesion studies observed by scanning electron microscope on the surface of CTS/TA at 50:50 (**a**), CTS/TA at 50:50 + 20% PEG (**b**), and CTS/TA at 50:50 + 20% PEG2 (**c**) where platelet attachment was reduced based on various material combination depicted

An assessment of platelet adhesion is a critical issue which has to be performed because it leads to activation of coagulation pathways eliciting blood clot formation (Fig. 3a–c). The addition of PEG1 and PEG2 caused no apparent platelet adhesion as compared to the chitosan/tannic acid films. Also, round morphology with no signs of activation with extended filopodia may be observed.

### Establishing cell cultures on the experimental films

**Fig. 4:**
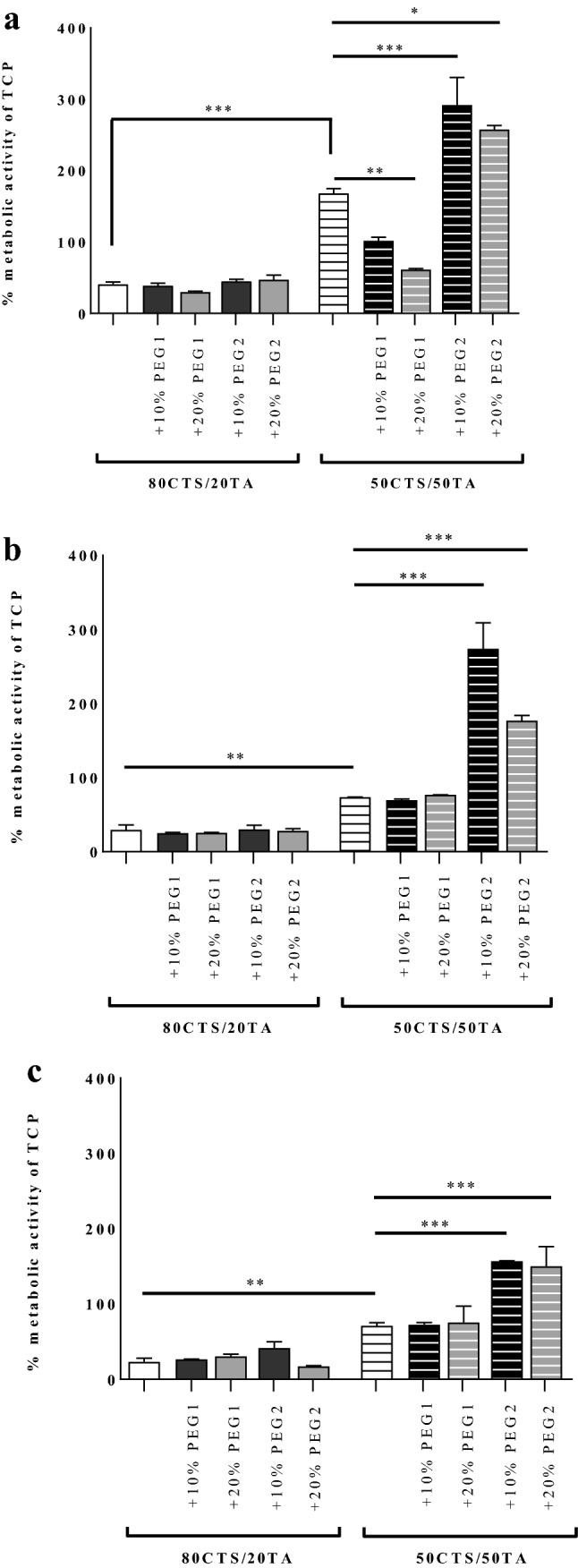
Metabolic activity of investigated materials; i.e. containing CTS/TA at 80:20 or CTS/TA at 50:50 with the additions of 10% or 20% of either PEG1 or PEG2 in human MNT-1 melanoma (**a**), human bone marrow mesenchymal stem cells (BMSC) (**b**) and human osteosarcoma (Saos-2) (**c**), and assessed as described in “Materials and methods”. Data were presented as the mean (*n* = 3) + SD. Statistically significant differences were indicated as *P < 0.05, **P < 0.01, ***P < 0.001

Tested cell lines investigated within this report revealed significant enhancement of metabolic activity of TCP compared of 5CTS/TA at 50:50 to CTS/TA at 80:20 alone for human MNT-1 melanoma, human bone marrow mesenchymal stem cells (BMSC) and human osteosarcoma (Saos-2) by 127% (*P* < 0.001; Fig. 4a), 45% (*P* < 0.01; Fig. 4b) and 48% (*P* < 0.01; Fig. 4c), respectively. These results indicate the higher cell compatibility with the increase of tannic acid content in the biomaterial. Furthermore, either PEG1- or PEG2-supplemented CTS/TA at 80:20 did not show any changes in cell viability what is in contrast to the increased content of TA, i.e. CTS/TA at 50:50. What is interesting, addition of PEG2, in both concentrations, such as 10% or 20%, revealed increased metabolic activity of TCP compared to CTS/TA at 50:50 alone by 123% (*P* < 0.001; Fig. 4a), 200% (*P* < 0.001; Fig. 4b), and 86% (*P* < 0.001; Fig. 4c) for CTS/TA at 50:50 + 10% PEG2 and by 89% (*P* < 0.01; Fig. 4a), 104% (*P* < 0.001; Fig. 4b), and 79% (*P* < 0.001; Fig. 4c) for CTS/TA at 50:50 + 20% PEG2.

## Discussion

The behavior of the films in the swelling state is an important factor for considering the material as a potential wound dressing since they are in direct contact with body fluids; therefore, they should be durable after soaking. We assume that only high-molecular-weight poly(ethylene glycol) improves the mechanical properties of chitosan/tannic acid-based films. Our results enclosed in this report and this statement are in line with Lima et al. (2018a; b) who noticed that the chitosan’s physical properties of the blends were improved by PEG addition depending on its molecular weight. Similarly, the correlation between PEG and its weight was noticed, i.e. the higher weight of investigated PEG, the increased mechanical parameters (Sun et al. 2008 ), and these observations are in line with our data, additionally confirmed by Xu et al. (2016). Altogether with previous reports, we claim that the higher PEG’s molecular weight is tested, e.g. ranging from 400 to 4000, the more molecular entanglements or electrostatic hindrance of mixture is seen. Next issue is about the optimal characteristics of biomaterial assessed regarding the wound management water vapor that plays the crucial role in efficacy boosting wound healing. Thus, WVPR is an essential factor for the materials to be considered as dressings since the moist environment of a wound is a highly desired condition to maintain the healing processes which is consistent with previous report showed by Michalska-Sionkowska et al. (2018). On the other hand, lower values of WVTR are responsible for accumulation of exudates which might retard the healing progress and increase the risk of bacterial contamination. The increasing amount of tannic acid is connected to the water vapor permeation rate. It is induced by “massive” presence of hydroxyl (–OH) groups in chemical formula which is able to bind water. It is desired that the moisture environment is more suitable for the wound healing; however, it may lead to the skin dehydration and scars formation (Morgado et al. 2015). On the one hand, we claim that increased amount of tannic acid correlates to enhanced WVPR while, on the other hand, the presence of PEG decreases this parameter. The reason for this was mentioned earlier, that the –OH functional groups within the formula of tannic acid triggering the ability to bind water in the environment. We know that PEG is soluble in water; however, as it is used as chitosan/tannic acid additive, it interacts with –OH groups of both compounds acting as cross-linkers by stabilizing the polymer structure. Thus, the presence of strong hydrogen bonds improves material stability; therefore, the mixture of CTS/TA together with PEG has different properties from that of pure PEG. Furthermore, the explanation for PEG-mediated dropped WVPR could be that poly(ethylene glycol) with high molecular weight presumably induces chains' conformation, subsequently structural changes, and finally affecting the WVPR.

Since the hemocompatibility is one of the key factors to consider material for potential biomedical application, we also assessed this parameter. As previously described by Weber et al. (2018), erythrocytes are sensitive to the hemolysis due to the shear stress and this is in line with our results where rate of erythrocytes hemolysis decreases proportionally with increasing concentration of both, PEG1 or PEG2. We compared our results with the ASTM F756-00 standard materials. Thus, hemolytic index ranging between 0 and 2% are considered as non-hemolytic while ranging 2–5% are slight hemolytic, and < 5% are classified as hemolytic ones (Pires et al. 2018). Similar results were obtained by Kameneva et al. (2003) where the PEG contact (any molecular weight) reduced the hemolysis rate of erythrocytes. Shih et al. (2011) also reported that PEG addition to the poly(hexa-methylene-urethane) resulted the lowering of hemolysis as the compatibility with blood is improved. This may suggest that the supplementation with PEG decreases of hemolysis rate indicating in parallel its high safety profile targeting for future application in regenerative medicine. Poly(ethylene glycol) improves material stability what was also proven earlier by Fu and Kao (2009). Thus, the released concentration of different compounds, e.g. silver sulfadiazine and bupivacaine hydrochloride, decreased with increasing amount of PEG added to gelatin acting as cross-linker. De Lima et al. (2018a; b) reported that the release of another phenolic acid–ferulic acid from chitosan-based material was rapidly released due to weak interactions between the phenolic acid and polymer, characterizing a burst effect. Such response was also detected in our experiments where burst effect was initially noticed with subsequent spontaneous increase of material degradation. The release of tannic acid from films obtained by our group showed decreases in concentration with increasing PEG amount. We claim that these differences are caused by the presence of interactions between PEG and tannic acid which inhibit the phenolic acid release from material. In fact, the influence of combinational content of PEG with chitosan on insulin released concentration was noticed by Sadhasivam et al. (2015) where the rate of insulin release was aided by presence of PEG. The addition of poly(ethylene glycol) resulted in repulsion to platelet adhesion and ensuing reduced platelet activation. This larger amount of resistance towards platelet attachment by water molecules can be attributed to the presence of a large number of hydrogen bonds between film components and water molecules. It has been previously reported by Deible et al. (1999) where platelet adhesion onto collagen was observed; however, the incorporation of PEG shows dramatic decrease in platelet adherence when contacted modified protein. This indicates that PEG is forming a type of coating which inhibits the platelets deposited onto the films surface. This observation was also reported by Sagnella and Mai-Ngam (2005) where PEG addition to the chitosan causes the significant decrease in platelet adhesion to the film as well as it decreases the density of surface charge.

Comparatively, our results with the ones obtained by the others listed above, it should be briefly mentioned that the optimal parameters of materials applied as dressings regarding the WVPR range from 1800 to 2300 g/m^2^/24 h (Xu et al. 2016) and hemolysis rate should not be higher as 5% (according to the ASTM F756-00 standard). In other words, as the WVPR is higher than the optimal conditions, there would be a need to moisture the wound surrounding; therefore, probability of the use of hydrogel form of material would be more desired. Similarly, the low platelet adhesion should be obtained in material contact with blood as it improves thromboresistance (Uchida et al. 2005). Indeed, dressing materials should not inhibit the proliferation of human cells; therefore, it is essential to study the cells’ behavior in contact with the designed material surface. Thus, our studies revealed that cells’ viability was significantly elevated compared to TCP, and this may suggest that materials proposed by us do not affect negatively the proliferation rate of cutaneous cells.

Besides, we supported our studies by cell assessments where our results are consistent with previous reports. For instance, chitosan grafting by PEG improved the L929 cells proliferation compared to materials without PEG (Mao et al. 2005). The presence of PEG in a mixture with chitosan influenced the breast cancer cells, which resulted in the inhibition of their proliferation (Chang et al. 2016), or finally PEG did not show any significant influence on the BMSC cells (Oda et al. 2014). These observations together with our results prompted us to make the statement that poly(ethylene glycol) may be also used in future for treatment of post-operation wounds in dermatology; however, further and prudent studies are strongly desired.

## Conclusion

The poly(ethylene glycol) addition may act as cross-linker for materials based on chitosan and tannic acid, and depending on the molecular weight of PEG their properties were changed. High-molecular-weight PEG improved mechanical properties of films, decreased the hemolysis rate, and it reduced the platelet adhesion to the material surface. Moreover, the addition of PEG1 or PEG2 in concentrations of either 10% or 20% to the materials containing CTS/TA at 80:20 does not affect cell viability in comparison to the control materials. On the other side, a statistically significant decrease of viability of MNT-1 cells was observed cultured on CTS/TA at 50:50 + 20% PEG1. Contrary, a prominent increase of viability was revealed for all types of cells cultured on the CTS/TA at 50:50 materials with the addition of both 10% and 20% PEG2. Chitosan/tannic acid thin films modified by PEGs may be potentially used as dressing materials, where those with high-molecular-weight PEG addition showed the inhibition of cancer cells viability and may be further studies for anticancer treatment.
